# Notes and News

**Published:** 1893-04-22

**Authors:** 


### Notes and News.

Collected and Communicated.

The North London Consumption Hospital will hold its annual festival dinner on Monday, 24th inst., at the Hotel Metropole, and H. L. W. Lawson, Esq., M.P., will preside. The new central block will be completed by Midsummer. This will provide accommodation for thirty-five extra patients, bringing the total number of beds up to eighty-four.

We have pointed out previously in our columns that influenza appears to be easily communicated by the medium of the post. In Paris at the present time the officials of the Post Office are suffering largely from the epidemic, so much so that the conduct of business is a serious difficulty. A good many epidemics are reported in France at the present time, amongst these being cholera, many cases being recorded from Lower Brittany.

A special appeal is being made for a convalescent fund in connection with St. Mary's Hospital. We are informed that a convalescent home, ready furnished, at Box Grove was placed at the disposal of the Convalescent Committee early last year, and was occupied for nearly seven months by the patients to their great advantage. The sum of £260 a-year is required to maintain it, and this will provide 113 patients with the inestimable benefit of three weeks' stay in the country to recruit their strength before returning to their homes. Without this sum the home must be given up. All those who have to do with the care of the sick know what a valuable adjunct to medical treatment the convalescent home is. As much as £200 a-year has been spent by the hospital for convalescent letters. The income of £260 required by the home the hospital can no longer afford. It is hoped that 520 annual subscribers of the small sum of 10s. will be found to solve the difficulty. Contributions will be gladly received for the committee by Miss E. Bird, at the hospital.

A minority of the hospital secretaries are, we understand, actively fermenting a strenuous opposition to the proposed Central Hospitals Board. It is reported that a meeting of these gentlemen has been held at the Royal Free Hospital, and that a member of the committee of one of the special hospitals has called a meeting of the opponents of the Central Board project to be held shortly at his private residence. There has been an unaccountable delay in the re-assembling of the enlarged committee charged with the duty of forming a scheme for the Central Hospitals Board. Lord Sandhurst returned from the Continent on Tuesday, and we hope he will lose no time in calling together the committee, so that this business of the Central Board may be disposed of finally one way or the other without further delay.

The Imperial Institute, which promises to afford a most imposing ceremony of national interest in May, is now almost entirely completed as far as the exterior is concerned. When the avenues of approach are finished and lined with trees, as is intended, the effect promises to be all that could be desired. The great hall will remain undecorated for the present, as the design eventually to be carried out will be too costly to carry out just yet. This will in no wise affect the useful nature of the building, and the requirements and comforts of those who use it will be well looked after.

The Hospital for Sick Children, Great Ormond Street, will not hold its annual festival this year. The new wing will be opened in June, and the committee are concentrating their energies in an effort to clear off the debt of £12,000 remaining on the new buildings. The hospital will now be able to give relief to an additional 800 children annually, and it is to be hoped that the executive will not be seriously hampered by want of means. A visit to the hospital, a walk through the comfortable and attractive wards, and a look into the children's cots could not fail to satisfy all as to the value of the work done in this institution. A pleasant addition to the Alexandra ward has been recently made in the shape of a charming American organ, presented by Mr. Walter Burns, whose name is associated with many kindly deeds.

Mr. H. W. Lawson, M.P., makes the following appeal on behalf of the North London Hospital for Consumption: "At the request of the Council of the North London Consumptive Hospital I have willingly consented to preside at the anniversary dinner in aid of its funds. Consumption and the kindred diseases of the chest are so rife that our hospitals are sorely taxed to cope with them. Well out of the reach of the fog demon, and standing in dry and bracing air, during the past thirty-three years this institution has been the means of relieving many thousands of patients who have been admitted from all parts of the United Kingdom. The increase of demand for admission is very large, and to meet it a new central block has been built, where thirty-five extra patients and the necessary working staff can be accommodated. May I ask you to help us, if only by a small contribution, to fight the disease which makes such havoc in this City and in this country."

